# Estimating number of European eel (*Anguilla anguilla*) individuals using environmental DNA and haplotype count in small rivers

**DOI:** 10.1002/ece3.9785

**Published:** 2023-02-26

**Authors:** Silje Halvorsen, Lars Korslund, Morten Mattingsdal, Audun Slettan

**Affiliations:** ^1^ Faculty of Engineering and Science University of Agder Kristiansand Norway

**Keywords:** *Anguilla anguilla*, conservation, environmental DNA, haplotype count, population quantification

## Abstract

Knowledge about population genetic data is important for effective conservation management. Genetic research traditionally requires sampling directly from the organism, for example tissue, which can be challenging, time‐consuming, and harmful to the animal. Environmental DNA (eDNA) approaches offer a way to sample genetic material noninvasively. In attempts to estimate population size of aquatic species using eDNA, researchers have found positive correlations between biomass and eDNA concentrations, but the approach is debated because of variations in the production and degrading of DNA in water. Recently, a more accurate eDNA‐approach has emerged, focusing on the genomic differences between individuals. In this study, we used eDNA from water samples to estimate the number of European eel (*Anguilla anguilla*) individuals by examining haplotypes in the mitochondrial D‐loop region, both in a closed aquatic environment with 10 eels of known haplotypes and in three rivers. The results revealed that it was possible to find every eel haplotype in the eDNA sample collected from the closed environment. We also found 13 unique haplotypes in the eDNA samples from the three rivers, which probably represent 13 eel individuals. This means that it is possible to obtain genomic information from European eel eDNA in water; however, more research is needed to develop the approach into a possible future tool for population quantification.

## Graphical abstract

We used eDNA from water samples to estimate the number of European eel (Anguilla anguilla) individuals by examining haplotypes in the mitochondrial D‐loop region, both in a closed aquatic environment with 10 eels of known haplotypes and in three rivers. The results revealed that it was possible to find every eel haplotype in the eDNA sample collected from the closed environment, and we also found 13 unique haplotypes in the eDNA samples from the three rivers, which probably represent 13 eel individuals.

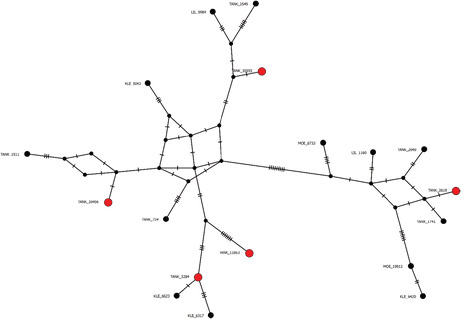

## 1 INTRODUCTION

The decline in global freshwater biodiversity is greater than in most affected terrestrial ecosystems (Grooten & Almond, 2018). Effective conservation management of species relies on population genetic data, which can be challenging to collect. Genetic research traditionally requires collecting biological samples from the organism of interest after using fishing and trapping methods, which can be stressful and cause harm, discomfort, or death (Bearzi, 2000; Romero & Reed, 2005). These approaches can also be time‐consuming and challenging when examining rare or elusive species (Jerde et al., 2011). Environmental DNA (eDNA) approaches offer a way to sample genetic material noninvasively, causing no significant damage to the species or the habitats (Antognazza et al., 2019), often being more sensitive and economically beneficial (Itakura et al., 2019; Thomsen & Willerslev, 2015), while also being capable of detecting rare and elusive species (Takahara et al., 2020).

There have been several attempts to examine the detection probabilities of eDNA where the conclusion often is that eDNA provides a snapshot of the species composition in space and time. In the sea, killer whale (*Orcinus orca*) eDNA are detected up to 2 h after individuals were observed in the sampled area (Baker et al., 2018) and eDNA from caged white trevally (*Pseudocaranx dentex*) are detected 30 m from the source (Murakami et al., 2019). In freshwater systems, eDNA from caged trout in fishless streams is detected 239.5 m downstream from the source (Jane et al., 2015).

In the effort to estimate species population size, positive correlations are found between, for example, eDNA concentration and biomass of common carp (*Cyprinnus carpio*) (Takahara et al., 2012) or eDNA concentration and biomass of eels (*Anguilla japonica*) (Itakura et al., 2019). However, the effectiveness of such approaches is debated because of variations in eDNA production and degradation rates (Lacoursiere‐Roussel et al., 2016), and several studies do not find significant correlations between observed biomass and eDNA quantity (Deutschmann et al., 2019). The explanation for the deviating results can be connected to the various factors affecting the eDNA concentration in water—both the shedding of eDNA by different species and individuals, the transport of eDNA in different environments, and the degrading of eDNA that varies with temperatures, microbial activities, and UV‐radiation (Shogren et al., 2017; Strickler et al., 2015), as well as different sampling methods.

However, a new approach focusing on the DNA sequence differences between individuals using eDNA is emerging. Uchii et al. (2016) estimated the degree of invasion of non‐native genotypes of common carp by eDNA, and Sigsgaard et al. (2016) used eDNA from seawater to study mitochondrial haplotypes of whale sharks (*Rhincodon typus*) and assessed the population structure. In addition, Parsons et al. (2018) developed an approach for generating population‐specific mitochondrial sequence data from eDNA using seawater samples, and Adams et al. (2022) recovered haplotypes from New Zealand blackfoot pāua (*Haliotis iris*) from marine eDNA samples. Concerning the challenges associated with eDNA concentration surveys, more accurate information about population size can be estimated by examining the DNA sequence differences between individuals. Determining the number of haplotypes in DNA‐regions with high genetic variability could be a tool for quantifying populations given sufficient genetic variation between individuals (Yoshitake et al., 2019). The D‐loop (regulatory) region in the mitochondrial genome is a variable area with intraspecific mutations (Sigsgaard et al., 2016). Recently, Yoshitake et al. (2019) examined haplotype diversity in Japanese eels (*Anguilla japonica*) and estimated the number of individuals in a population by sequencing the mitochondrial D‐loop region. It has been found that the D‐loop region of the related species European eel (*Anguilla anguilla*) has a haplotype diversity of h = 0.995 (0.996 in the North Sea) (Ragauskas et al., 2014), which means that almost every individual has a unique DNA sequence in this region. Studying this DNA region is therefore suitable when aiming to distinguish between haplotypes and thus counting individuals in the area where water samples are collected.

Anguillid eels are catadromous and inhabit rivers, lakes, brackish water, the coast, and the sea (Thorstad et al., 2010). The European eel is currently labeled as critically endangered by the International Union for Conservation of Nature (IUCN) Red List (Pike & Gollock, 2020) and the Norwegian Red List. The species is affected by threats at numerous developmental phases of its complex life, including overfishing, illegal trade and aquaculture (Castonguay et al., 1994; Shiraishi & Crook, 2015), habitat loss and destruction (Halvorsen et al., 2020; Kettle et al., 2011), freshwater parasites (Feunteun, 2002), poisoning (Belpaire et al., 2009) in addition to ocean changes and global warming (Drouineau et al., 2018; Friedland et al., 2007). Despite conservation efforts by EU member countries in response to the European Commission Regulation EC 110/2007 (ICES, 2019), the stock is currently decreasing (Pike & Gollock, 2020), and protection of the species is needed.

In this study, we aim to use eDNA from water samples in an effort to estimate the number of *A. anguilla* individuals by examining haplotypes in the mitochondrial D‐loop region and to experimentally examine whether eDNA‐haplotype information collected from a water sample is compliant with the genomic haplotype information obtained from tissue samples of each individual. Second, we aim to examine how many different *A. anguilla* haplotypes we can find at selected locations in three different rivers.

## 2 MATERIALS AND METHODS

### 2.1 Genomic DNA and eDNA from 10 eel individuals

Ten eels were caught by electrofishing in the river Lilleelv, September 20, 2018. A tissue sample of each individual was gathered by a small fin‐clip from the caudal fin and preserved in 96% ethanol. We then transferred every individual to an 80‐L tank with approximately 60 L of well‐oxygenated water from the river. After the 10 eels had been in the tank for 1 h, without any water replacement, a water sample from the tank (for now on referred to as TANK) was collected, and from the river itself (see description below), before the eels were released back into the river.

### 2.2 Water sampling

We sampled water in the rivers Kleplandsbekken (for now on referred to as KLE, 58,1045°N. 7,8232°E) and Moelva (for now on referred to as MOE, 58,2552°N 8,3881°E) in June 2018 and Lilleelv (for now on referred to as LIL, 58,4429°N. 8,6908°E) in September 2018 in the county of Agder, in South Norway. The sampling and processing of samples followed method described by Halvorsen et al. (2020). Each sample consisted of 1 L surface water, which was stored on ice until filtration (within 5 h). New gloves were used for each sampling, and the bottles were rinsed in 10% chlorine followed by tap water before each sampling. Back in the laboratory, 300–1000 mL water (as much as possible) was filtrated through a 0.45 μm pore size cellulose nitrate filter (Thermo Scientific Nalgene) by an ILMVAC vacuum pump (GmbH). The filters were folded and stored at −20°C after filtration.

### 2.3 DNA extraction

We used DNeasy® Blood and Tissue Kit (Qiagen) and bead beating to extract DNA from water samples using a method described by Thomsen et al. (2012). The same kit was used to extract DNA from the tissue samples following the protocol of the producer (Qiagen). After isolation, the eDNA was stored in microcentrifuge tubes at −20°C. We performed the eDNA isolation in a separate room from the PCR amplification. Every sample was analyzed by a spectrophotometer (NanoDrop™ One, Thermo Scientific) after isolation to examine purity and eDNA‐concentration.

### 2.4 PCR amplification

We amplified a 731 bp section of the mitochondrial D‐loop region from *A. anguilla* from every DNA sample using PCR and specific primers (Table 1). The PCR products from the eDNA‐samples were additionally amplified by a nested PCR with nested primers (Table 1) to improve sensitivity and specificity. The primers were designed with Primer‐BLAST at the web page of the National Center for Biotechnology Information (NCBI) and the program Primer Express 3.0.1 (Thermo Fisher). The primers were tested for species‐specificity by searching for homology to DNA sequences from species that could be found in the same area using Clustal Omega (European Bioinformatics Institute) and NCBI's GenBank. In order to avoid false haplotypes caused by erroneous inserted nucleotides in the PCR, a high‐fidelity DNA polymerase was used.

**TABLE 1:** Primers and nested primers in 5′ to 3′ direction for the sequence in the D‐loop region for *A. anguilla* (731 bp product).

Primers	AaD‐F: CCTAGCGCTAAAAATCAGAGAGG AaD‐R: TGGCAAACTTTTTAGAAGGTGTCT
Nested primers	**AaDN‐F:** CGCTAAAAATCAGAGAGGAAAGATTT **AaDN‐R:** ACTTTTTAGAAGGTGTCTCACATGTAA

The PCR‐mix had the following ingredient concentrations: 1 x Phusion Green Hot Start II High‐Fidelity PCR Master Mix (Thermo Fisher), 0.9 μM AaD‐F, and 0.9 μM AaD‐R. Fifteen microliters PCR mix with 5 μL eDNA‐template or 19 μL PCR mix with 1 μL genomic DNA template was transferred to a 0.1 mL Micro Fast Tube Strip (Thermo Fisher). We conducted PCR in a Veriti 96 Well Thermal Cycler PCR System (Applied Biosystem). The thermal condition of the PCR for the genomic DNA samples was as follows: 1 incubation of 98°C in 3 min, 40 cycles of 98°C in 3 s, 59°C in 30 s, 72°C in 30 s, and 1 incubation of 72°C in 5 m, and the thermal condition for the eDNA‐samples was as follows: 1 incubation of 95°C in 5 min, 40 cycles of 95°C in 15 s, 57°C in 15 s, 72°C in 30 s, and 1 incubation of 72°C in 7 m. PCR‐grade H_2_O was used as template in a negative control reaction, and genomic DNA from *A. anguilla* was used as template in a positive control reaction. eDNA samples were amplified by nested PCR to increase the specificity and secure enough product for sequencing. The PCR mix had the same ingredient concentrations as described above, but with 1 μL of the PCR‐products instead of the eDNA‐templates, and the nested primers AaDN‐F and AaDN‐R replaced the original primers (Table 1, Appendix A). The nested PCR had the same temperature profile, but the initial denaturation step of 95°C lasted 10 min instead of 5 min.

Following amplification, we transferred 10 μL PCR product to a 1% agarose gel for electrophoresis in 30 m at 90 V to confirm a successful amplification.

### 2.5 Preparation and sequencing

The PCR‐templates were purified by PureLink® Quick Gel Extraction and PCR Purification Combo Kit (Invitrogen) following the producer's protocol and quantified using the Qubit dsDNA broad‐range assay (Invitrogen). The amplicons from genomic DNA were Sanger sequenced by Eurofins Scientific (Ebersberg, Germany). The amplicons from eDNA‐samples were pair‐end sequenced (2 × 250 bp) with the Illumina MiSeq platform by Norwegian Sequencing Centre (Oslo, Norway).

### 2.6 Analysis of Illumina sequencing data

We used cutadapt (Martin, 2011) to remove primers and nested primers. We used DADA2 to determine haplotype variants using denoising (Callahan et al., 2016). Briefly, the denoising algorithm estimates the sample‐specific error rates for every possible nucleotide transversions and transition from the data and infers the sequence composition of the samples after convergence of the algorithm. In both methods, we used the default values (Tsuji, Miya, et al., 2020). In DADA2, we defined the following parameters when filtering reads and learning error rates: default expected error rate (maxEE = 2), minimum read length to 250 (minLen = 250), and truncated reads if any base had a quality score of 2 or less (truncQ = 2), and subsequently removed any truncated reads less than 250 (truncLen = 250). As the reads had mixed‐orientation, the option “orient.fwd” was used specifying the five first bases of the forward primer. Chimeras were removed and the sequence table was constructed.

The effect of sequencing errors and false haplotypes can be mitigated by assessing sequence abundance per haplotype. We ignored haplotypes with less than 1% of total reads per sample (Tsuji, Maruyama, et al., 2020).

The four samples were processed simultaneously. The reads were filtered and trimmed using default settings. Approximately ~40% of the river data did not contain the nested primer and was excluded. Then, error rates were estimated using the learnErrors function and reads were corrected and assigned to the representative sequence community. Then, the forward and reverse reads were merged using mergePairs. As the primer design dot does not allow for overlapping reads, the justConcatenate option was set to true. Finally, chimeras were identified using the pooled strategy and removed. Here, the TANK sample contained 54% chimeras which were excluded.

### 2.7 Sequence analysis

Sequences were aligned using default MAFFT (Katoh et al., 2002) and visualized in PopArt (Leigh & Bryant, 2015) using median joining. We also obtained 56 complete *Anguilla anguilla* mitogenomes (Jacobsen et al., 2014). Again, we aligned these using default MAFFT settings and edited the resulting alignment in JalView (Waterhouse et al., 2009) to remove gapped regions introduced by our study, leaving the D‐region. We did not include sequences from Ragauskas et al. (2014) as our reverse sequences did not overlap with their reported sequences.

## 3 RESULTS

### 3.1 Sequencing data

In total, there were 592,761 reads, from the LIL (103,788), MOE (112,289), KLE (99,289) rivers, and the TANK (277,395) sample. After filtering and cleaning the data, 26,645 (MOE), 21,514 (LIL), 24,403 (KLE), and 46,341 (TANK) reads survived and were assigned to one of the 1059 representative sequences. Of these, 1059 representative sequences did 23 capture 99% of all quality‐controlled reads.

### 3.2 Haplotypes observed in the tank

**FIGURE 1:**
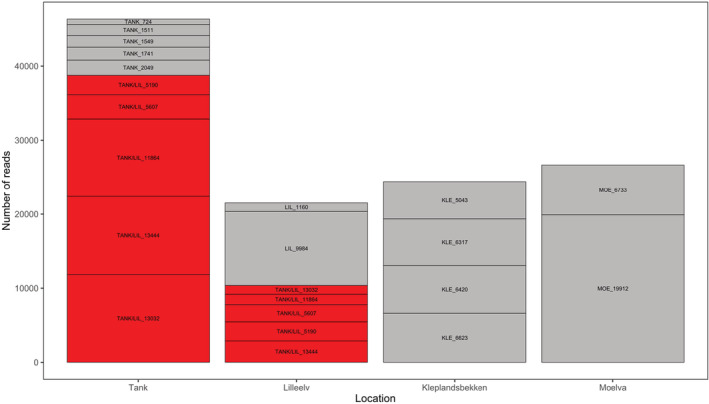
Detection of *Anguilla anguilla* haplotypes from four eDNA samples (tank with 10 eel individuals, and the rivers Lilleelv, Kleplandsbekken, and Moelva). Y‐axis shows the number of reads assigned to the haplotypes. Each segment of a stacked bar represents a haplotype, and the height of the segment is proportional to the number of reads of that sample. The haplotype notation is “site”_“total number of reads” Ten unique haplotypes were observed in the tank (TANK), seven in the river Lilleelv (LIL), four in Kleplandsbekken (KLE), and two in Moelva (MOE). The five haplotypes that were detected both in the tank and in the Lilleelv sample are colored red.

Ten haplotypes were detected in the tank water, where 10 eel individuals from the river Lilleelv had been kept (Figure 1). Each of the 10 haplotypes had an identical match to the haplotype detected in the genomic DNA sample from each of the 10 eel‐individuals.

### 3.3 Haplotypes observed in the rivers

**FIGURE 2:**
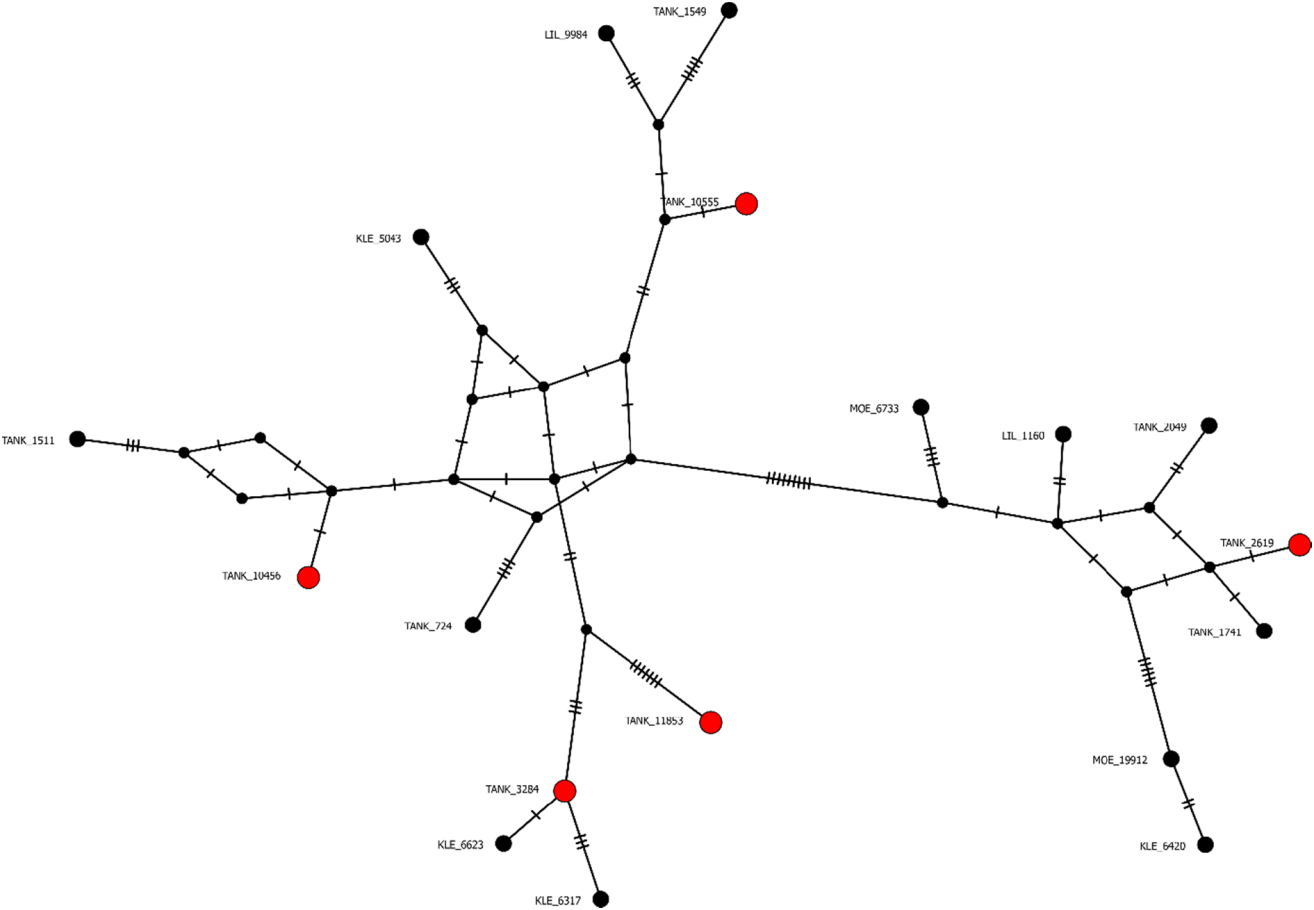
Haplotype network with the inferred and aligned sequences. Colors and notations are similar to that of Figure 1, and ticks on edges represent sequence changes.

In total, 18 eel haplotypes were detected in the three water samples from rivers. Seven were detected in the eDNA sample from the river Lilleelv, and of these seven, five had an identical match among the 10 haplotypes found in the tank water (see haplotypes colored red in Figures 1 and 2). Four *A. anguilla* haplotypes were detected in the eDNA sample from Kleplandsbekken, and two in Moelva.

### 3.4 Sequence analysis

Aligning detected D‐region sequences against previously published mitogenomes revealed 81 segregating sites, 52 parsimony‐informative sites, and a nucleotide diversity *n* = 0.024 (for access to dataset, see Mattingsdal (2022)). The alignment of the 18 unique haplotypes detected in the water sample from each of the three the rivers and in the water sample from the tank showed that there is no obvious genetic structure in the population. Diversity is high and haplotypes originating from the same river are not grouped together (see haplotype network in Figure 2). Of the 18 unique haplotypes identified, four were found to be identical to four of the 56 previously described *Anguilla anguilla* mitogenomes (Jacobsen et al., 2014).

## 4 DISCUSSION

### 4.1 Haplotype count

By examining eDNA from water samples, we were able to differentiate between *A. anguilla* haplotypes in the mitochondrial D‐loop region. In the three rivers Lilleelv, Kleplandsbekken, and Moelva, we found seven, four, and two unique haplotypes, respectively, which probably represent different eel individuals. In addition, it was possible to identify every 10 eel individuals in the tank water by analyzing eDNA from a tank water sample and comparing it with the genomic haplotype information obtained from tissue samples of the same 10 eel individuals. This supports earlier studies that eDNA haplotyping of individual species in water samples is as effective as tissue sampling (Dugal et al., 2021). The *Anguilla* genus is highly diverse in the mitochondrial D‐loop region (Ragauskas et al., 2014), and our method is therefore particularly feasible for this species. If studying other species with lower haplotype diversity, such as fish species with kinship in the same river system, one might need to examine nuclear DNA (nDNA) to distinguish between individuals. Because of higher effective population size of nuclear genome than mitochondria genome and the possibility to include higher number of loci/haplotypes using nuclear DNA markers, it might be favorable to analyze nDNA in such cases. However, the high copy number of mitochondrial genome compared with nDNA in cells increases the possibility of detection in eDNA analyses. Development of long‐read sequencing approaches and nDNA markers would be beneficial for population genetic studies (Adams et al., 2019).

### 4.2 Detectable eDNA after removal of eel individuals

Haplotypes from five of the 10 individuals in the tank water were also detected in the river water sample, which means that eDNA from these five individuals were detectable in the river one hour after they were caught and removed from their environment. Given that this is running water and that the discharge was about 0.48 m^3^/second (i.e., 1720 m^3^/hour) at the time of sampling, it is interesting to find that DNA from these individuals was still detectable one hour later. Various studies have shown that eDNA can be detected in standing water days after the source of the DNA has been removed, see, for example, Barnes et al. (2014), but in running water samples of several liters has often been necessary to compensate for the reduced probability of detection caused by removal of eDNA by water flow (Rees et al., 2014). Field experiments in rivers with low discharge (<100 liters/second) has shown that eDNA concentrations are relatively stable the first 24 h after the source of DNA has been removed, but that eDNA concentration decreases with increased discharge (Jane et al., 2015; Nevers et al., 2020). The discharge when we collected the sample was much higher, and this should have reduced the probability of detection even more. However, in aquatic environments, eDNA is found to easily bind to the sediment, which has the capability to store eDNA for days or weeks (Sakata et al., 2020; Strickler et al., 2015; Turner et al., 2015; Wei et al., 2018). The bottom substrate at the sample site in Lilleelv consists of mainly silt, sand, and some gravel, and it is likely that eDNA <>stored in this sediment was continuously released into the flowing water and detected in our sample 1 h after removal of the individual eels, despite the higher discharge. The five haplotypes found in both the river and the tank sample were the five that were most abundant in the tank sample. This might indicate that these are originating from individuals that, for some reason, shed more eDNA into the surrounding than the average individual does. If so, it is not surprising that eDNA from these five were the still present in the river water 1 h later.

### 4.3 Eel haplotype diversity and detection rates

Of the 18 unique haplotypes detected, four haplotypes were identical in the D‐loop region to four of 56 previously described haplotypes (Jacobsen et al., 2014). This suggests that the haplotype diversity could be lower than previously described (h = 0.996 in the North Sea) (Ragauskas et al., 2014). Two of the four haplotypes that were identical to previously described haplotypes pertained to eels in Lilleelv and the tank which were Sanger‐sequenced, but the other two were only Illumina‐sequenced. The PCR products were 730 bp, but the Illumina pair‐end sequencing only covered 250 bp from each end. That is, the forward and reverse read in the Illumina sequencing did not overlap, which means that there is a section in the middle of the amplicon that is excluded. This section may inhabit nucleotide variations that could distinguish the two Illumina‐sequenced haplotypes from the previously described haplotypes they seem identical to. In addition, the illustration of our haplotype network (Figure 2) shows that there is no sign of genetic similarities between individuals in the rivers.

The analysis of the sequence data revealed that one of the haplotypes (in the river Moelva, Figure 1) had a much higher detection rate than other haplotypes from the river samples. The explanation can be spatial heterogeneity of eDNA: The water sample might have been collected close to, in space and time, the individual, leading to a high number of reads. It is also possible that one individual was larger or more active than the others, and shed more eDNA to the water. Regardless of the cause, this is not affecting the total number of unique haplotypes detected, and therefore neither the inferred number of individuals at the site. If we had studied eDNA‐concentration per se in order to estimate the number of eel individuals, these factors could easily have led to misleading results. This supports the suggestion that population structure estimates based on eDNA from water samples should origin from the presence/absence of haplotypes (Azarian et al., 2021). In addition, a high copy number of one haplotype would also be observed if two individuals share the same haplotype. That would cause an underestimation of the number of individuals. However, the probability of two eel individuals sharing a haplotype of the studied D‐loop region is low considering the estimates of Ragauskas et al. (2014). Even if haplotype diversity is somewhat lower, our approach will still provide an estimate of the minimum number of individuals present, as we can be certain that there must at least be as many individuals present as haplotypes detected. If we assume equal haplotype diversity in time and space, this will still be a valuable way to estimate the minimum number of individuals present. The phenomenon heteroplasmy, which is the presence of more than one mitochondrial haplotype within a cell or individual, could theoretically influence our estimates of number of individuals at a location. However, the frequency of the extra haplotype in heteroplasmy is in general low and will probably not influence these estimates notably.

### 4.4 Challenges with the study

One of the challenges with this approach is the error rates in PCR and sequencing, which can be mistaken as natural mutations in the sequences. To secure the most accurate results, we used High‐Fidelity DNA polymerase in the PCR. In the analysis of our sequences, we found that the natural difference between haplotypes of eel generally was larger than expected PCR and sequencing errors.

Determining the set of representative sequences (OTUs) is nontrivial and can be achieved by clustering, denoising, or both (Antich et al., 2021; Brandt et al., 2021). However, determining which OTUs to consider true or false remains somewhat challenging, as often hundreds of OTUs are identified, in which some have a very low abundance. Informed by our experimental setup, we set the lower threshold to the rate in which all known individuals were recalled (valid OTU >1% abundance), and applied that threshold to the samples from the field, similarly as Tsuji, Miya, et al. (2020). The same values were then applied to the rivers. In this case, 99% of all reads were included, while the remaining 1% were counted as sequencing errors. The output of the sequencing analysis, and consequently the haplotype estimates, is of course sensitive to the cutoff values that are set. However, in this study, the number of haplotypes found in the rivers was the same with a cutoff value of 1% or 5%, indicating that the conclusions are robust to the chosen cutoff and that real haplotypes can likely be distinguished from false haplotypes (sequencing errors) based on large relative number of reads. Still, using consistent cutoff values when studying a species over time would likely give the most accurate and consistent haplotype estimates.

A potential weakness of the study could be the lack of field and extraction controls. Ideally, we should have brought with us distilled water in field, or collected water samples in a lake with guaranteed absence of eels, and treated them the same way as the water samples. However, every water sample we collected was sequenced, and a contamination between water samples should therefore be disclosed as one haplotype detected in more than one river sample. No haplotype was found in more than one river, and this indicates that there have been no cross contaminations between water samples during filed or lab work.

Considering the estimated number of eels in the three rivers, we are not able to define the size of the area which the water samples cover, and thus which part of the river the individuals pertain to. Further work would be to collect a sufficient number of samples along a river to detect the highest number of haplotypes possible, and to see to what extent the haplotype signatures is allocated in space (e.g., along a river or within a lake).

## 5 CONCLUSIONS

By examining the mitochondrial D‐loop region in eDNA samples collected from three rivers, we found 18 unique *A. anguilla* haplotypes. Because of the high haplotype diversity of the species, the haplotypes probably represent 18 individuals. In a closed environment with 10 eels, we also found that the eDNA haplotype information collected from the water sample was compliant with the D‐loop haplotype information obtained from tissue samples of each individual. Our results reveal that it is possible to obtain haplotype information from European eel eDNA in water, which could be the initial phase of a possible future quantification method for this species, but also for other aquatic species. However, more research is needed to develop the approach into a future tool for population quantitation.

## AUTHOR CONTRIBUTIONS

**Silje Halvorsen:** Conceptualization (equal); investigation (equal); methodology (equal); project administration (lead); resources (lead); writing – original draft (lead); writing – review and editing (lead). **Lars Mørch Korslund:** Formal analysis (equal); supervision (supporting); writing – review and editing (supporting). **Morten Mattingsdal:** Data curation (lead); formal analysis (equal); writing – review and editing (supporting). **Audun Slettan:** Conceptualization (equal); methodology (supporting); project administration (equal); supervision (equal); writing – review and editing (supporting).

## CONFLICT OF INTEREST STATEMENT

We have no conflicts of interest to declare.

## Data Availability

The data that support the findings of this study are openly available in Figshare at https://doi.org/10.6084/m9.figshare.19208214.v1.

## APPENDIX A

Primers AaDN‐F and AaDN‐R compared with mtDNA from species possible to find at the locations where water samples were collected. The sequences are the best match between the primers and the mtDNA from the other fish species. Hyphens “‐” in the sequences represent identical base pairs. The comparisons were conducted with Clustal Omega, and the sequences were found in the NCBI's GenBank.

**Table:**

*Salmo trutta* GenBank: LC137015.1	AaDN‐F *S.trutta*	CGCTAAAAATCAGAGAGGAAAGATTT ‐‐G‐T‐‐‐G‐‐C‐‐‐CA‐G‐‐TTA‐G
	AaDN‐R *S.trutta*	TTACATGTGAGACACCTTCTAAAAAGTC‐‐AC‐C‐‐‐TT‐T‐‐‐‐‐‐GTG‐G‐‐
*Salvelinus alpinus* GenBank: MF621743.1	AaDN‐F *S.alpinus*	CGCTAAAAATCAGAGAGGAAAGATTT‐C‐‐‐GTGC‐‐‐‐‐‐‐‐AGG‐‐‐‐‐‐
	AaDN‐R *S.alpinus*	TTACATGTGAGACACCTTCTAAAAAGT‐‐‐AC‐T‐A‐A‐A‐‐‐‐‐A‐‐‐TGCC‐
*Lampreta fluviatilis* GenBank: FP929026.1	AaDN‐F *L.fluv*.	CGCTAAAAATCAGAGAGGAAAGATTT‐‐T‐‐C‐‐‐‐TCT‐C‐‐C‐GCA‐‐‐‐
	AaDN‐R *L.fluv*.	TTACATGTGAGACACCTTCTAAAAAGT GC–GC‐‐A‐‐T‐A‐A‐G‐T‐‐‐‐‐T‐‐
*Phoxinus phoxinus* GenBank: AB671170.1	AaDN‐F *P. phoxinus*	CGCTAAAAATCAGAGAGGAAAGATTT ‐‐T‐‐‐‐GC‐‐G‐‐C‐‐A‐‐GA‐‐‐‐
	AaDN‐R *P. phoxinus*	TTACATGTGAGACACCTTCTAAAAAGTA‐GA‐‐C‐‐‐‐G‐GG‐‐‐‐‐C‐GT‐‐A
*Coregonus lavaretus* GenBank: AB034824.1	AaDN‐F *C. lavaretus*	CGCTAAAAATCAGAGAGGAAAGATTT‐C‐‐‐GTGC‐‐‐‐‐‐‐‐AGG‐‐‐‐‐‐
	AaDN‐R *C. lavaretus*	TTACATGTGAGACACCTTCTAAAAAGTC‐‐GC‐A‐‐G‐‐A‐TA‐‐‐‐T–T‐CT‐
*Osmerus eperlanus* GenBank: MH238073.1	AaDN‐F *O. eperlanus*	CGCTAAAAATCAGAGAGGAAAGATTT ‐‐T‐‐‐‐GC‐‐‐‐‐C‐‐C‐C‐‐‐CC‐
	AaDN‐R *O. eperlanus*	TTACATGTGAGACACCTTCTAAAAAGTA‐C‐TA‐‐‐T‐G‐‐GGA‐‐‐GG‐‐‐‐‐
*Perca fluviatilis* GenBank: KM410088.1	AaDN‐F *P. fluv*	CGCTAAAAATCAGAGAGGAAAGATTTA‐TG‐‐GCC‐A‐A‐A‐‐C‐G‐‐‐‐‐‐
	AaDN‐R *P. fluv*.	TTACATGTGAGACACCTTCTAAAAAGTA‐GG‐‐C‐‐‐‐G‐GG‐‐‐‐‐C‐GT‐‐A
